# The NF-κB signalling pathway and TM7SF3 contribute to liver fibrosis caused by secreted phospholipase A2 of *Clonorchis sinensis*

**DOI:** 10.1186/s13071-021-04663-z

**Published:** 2021-03-10

**Authors:** Yin-Juan Wu, Qing He, Mei Shang, Ying-Xuan Yin, Ye Li, Xue Du, Xue-Rong Li

**Affiliations:** 1 Department of Parasitology, Zhongshan School of Medicine, Sun Yat-sen University, Guangzhou, Guangdong 510080 People’s Republic of China; 2 Key Laboratory for Tropical Diseases Control of Ministry of Education, Sun Yat-sen University, Guangzhou, Guangdong 510080 People’s Republic of China; 3 Provincial Engineering Technology Research Center for Biological Vector Control, Guangzhou, Guangdong 510080 People’s Republic of China; 4 Department of Clinical Laboratory, the Third Affiliated Hospital of Sun Yat-sen University, Guangzhou, Guangdong 510630 People’s Republic of China

**Keywords:** Pathogenesis, *Clonorchis sinensis*, Liver fibrosis, Phospholipase A2, Receptor

## Abstract

**Background:**

The NF-κB signalling pathway has been reported to be related to liver fibrosis, and we investigated whether the NF-κB signalling pathway is involved in liver fibrosis caused by secreted phospholipase A2 of *Clonorchis sinensis* (*Cs*sPLA2). Furthermore, expression of the receptor of *Cs*sPLA2 on the cell surface of hepatic stellate cells (HSCs) may greatly contribute to liver fibrosis.

**Methods:**

*Cs*sPLA2 was administered to BALB/c mice by abdominal injection. The levels of markers of NF-κB signalling pathway activation in mouse liver tissue were measured by quantitative RT-PCR, ELISA and western blot. Additionally, HSCs were incubated with *Cs*sPLA2, and an NF-κB signalling inhibitor (BAY 11-7082) was applied to test whether the NF-κB signalling pathway plays a role in the effect of *Cs*sPLA2. Then, the interaction between *Cs*sPLA2 and its receptor transmembrane 7 superfamily member 3 (TM7SF3) was confirmed by co-immunoprecipitation (co-IP) and GST pull-down. To determine how TM7SF3 influences the ability of *Cs*sPLA2 to cause liver fibrosis, a TM7SF3 antibody was used to block TM7SF3.

**Results:**

The levels of the NF-ΚB signalling pathway activation markers TNF-α, IL-1β and phospho-p65 were increased by *Cs*sPLA2 in the context of liver fibrosis. In addition, the interaction between TM7SF3 and *Cs*sPLA2 was confirmed by co-IP and GST pull-down. When TM7SF3 was blocked by an antibody targeting 1–295 amino acids of TM7SF3, activation of HSCs caused by *Cs*sPLA2 was alleviated.

**Conclusions:**

The NF-ΚB signalling pathway is involved in the activation of HSCs by *Cs*sPLA2. TM7SF3, the receptor of *Cs*sPLA2, plays important roles in liver fibrosis caused by *Cs*sPLA2.
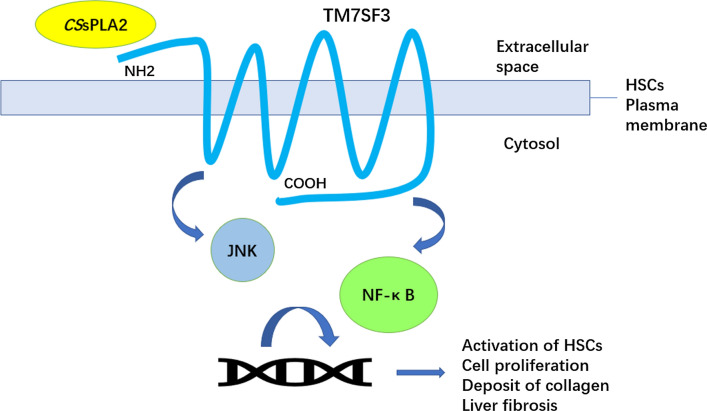

## Background

*Clonorchis sinensis* is a common parasite present mainly in East and South Asian countries, such as China, Korea and Vietnam [1, 2]. Infection of *Clonorchis sinensis* can cause cholangitis, cholecystitis and cholelithiasis and, in some serious cases, liver fibrosis or even cholangiocarcinoma [3]. Deposition of a large amount of collagen caused by the activation of hepatic stellate cells (HSCs) is key to liver fibrosis caused by infection with *Clonorchis sinensis* [4]. However, the mechanism by which HSCs are activated is still unclear. In our previous research, it was proven that *Cs*sPLA2 can activate HSCs and result in liver fibrosis through the activation of the c-Jun N-terminal kinase (JNK) signalling pathway [5]. However, it is still unknown whether *Cs*sPLA2 can activate HSCs through other cell signalling pathways. Cytokines such as TNF-α and IL-1β have been found to be associated with the activation of HSCs in many reports [6]. The cytokines are related to the NF-κB signalling pathway and are released when this pathway is activated [7]. It is possible that *Cs*sPLA2, an enzyme itself and member of the group III phospholipase family, can activate HSCs through the NF-κB signalling pathway. However, the mechanism by which *Cs*sPLA2 causes liver fibrosis is associated with cell signalling pathways rather than its enzymatic activity [5]. Activation of cell signalling pathways is thought to be caused by the binding of a protein to its receptor on the surface of the cell [8–10]. In our previous research, TM7SF3, a transmembrane protein [11–13] and receptor of *Cs*sPLA2, was screened by a yeast two-hybrid system. TM7SF3 contains seven putative transmembrane domains, and its open reading frame encodes a protein of 570 amino acids. In this research, the ability of *Cs*sPLA2 to bind TM7SF3 is tested, and we investigate how TM7SF3 influences the ability of *Cs*sPLA2 to cause liver fibrosis.

The excretory secretory products (ESPs) of parasites play important roles in host–parasite interactions and the pathogenesis of diseases, including immunosuppression, fibrosis and carcinoma [14]. The ESPs of *Clonorchis sinensis,* which include 110 proteins, such as glycometabolic enzymes, detoxification enzymes and a number of RAB family proteins, are closely related to biliary diseases and liver fibrosis [15].

*Cs*sPLA2 is one of these ESPs. The gene that encodes *Cs*sPLA2 contains 828 bp. *Cs*sPLA2 is a 34-kDa protein, and we previously purified a recombinant MBP-*Cs*sPLA2 protein with a molecular weight of 76 kDa. *Cs*sPLA2 is a group III PLA2 and has been determined to cause liver fibrosis [16, 17]. It is being considered as a potential drug target [16]. It has been proven that this protein is able to bind to the HSC membrane and cause upregulation of collagen expression, which can result in liver fibrosis [16]. TM7SF3, the receptor of *Cs*sPLA2, is expressed on the membranes of HSCs and may play important roles in liver fibrosis caused by *Cs*sPLA2. The NF-κB signalling pathway has been reported to be associated with liver fibrosis [18]; therefore, it is necessary to investigate the relationship between liver fibrosis caused by *Cs*sPLA2 and this signalling pathway.

## Methods

### Expression and purification of MBP-*Cs*sPLA2 protein

The recombinant plasmids (pMAL-c2x-*Cs*sPLA2) were transformed to *E. coli* BL21 (DE3). Overnight cultures carrying the recombinant plasmid pMAL-c2x-*Cs*sPLA2 were inoculated in 2 L of Luria–Bertani broth medium containing 50 μg/ml of ampicillin. Cells were grown at 37 °C until the A600 had reached 0.6. The expression of protein was induced by adding IPTG at a final concentration of 0.5 mM, followed by incubation at 37 °C for 4 h with vigorous shaking at 250 rpm. MBP-*Cs*sPLA2 protein was purified by a single step of amylose resin under native conditions (NEB, USA), following the manufacturer’s protocol. The endotoxin in the recombinant protein was removed by Detoxi-Gel™ Endotoxin Removing Gel (Thermo, USA), and then detected by limulus test, which confirmed that no endotoxin was found in the recombinant protein. The recombinant protein was stored at −80 °C for later use.

### Measurement of the levels of NF-κB signalling pathway activation markers in mouse liver tissues

Male BALB/c mice aged 8 weeks and weighing approximately 19–20 g were raised in a specific pathogen-free (SPF) environment. They were divided into three groups with three mice in each group. The three groups of Balb/c mice were given an abdominal injection of 100 μg PBS, 100 μg maltose-binding protein (MBP), or 100 μg MBP-*Cs*sPLA2, twice a week for 4 weeks. The mice were sacrificed after 4 weeks.

Total RNA was extracted from mouse liver tissue by TRIzol (Invitrogen, USA). cDNA was obtained by reverse transcription from one microgram of total RNA with a reverse transcriptase kit (Thermo, Lithuania). SYBR Premix ExTaq (Takara, China) was used for PCR amplification, and the transcribed cDNA was used as a template. The sequences of the primers for mouse TNF-α were 5′-CAT CCT CTC AAA ATT CGA GTGACA-3′ (forward primer) and 5′-TGG GAG TAG ACA AGG TAC AAC CC-3′ (reverse primer). The sequences of the primers for mouse IL-1β were 5′-AAA TGC CAC CTT TTG ACA GTG ATG-3′ (forward primer) and 5′-GCT CTT GTT GAT GTG CTG CTG-3′ (reverse primer). The sequences of the primers for mouse glyceraldehyde-3-phosphate dehydrogenase (GAPDH) were 5′-CAA AAT GGT GAA GGT CGG TGT G-3′ (forward primer) and 5′-TGA TGT TAG TGG GGT CTG GCT C-3′ (reverse primer). Mouse GAPDH was used as the internal standard for normalization. The PCR conditions were as follows: 95 °C for 30 s followed by 40 cycles of 95 °C for 5 s, and 60 °C for 30 s, with an incremental increase of 0.5 °C for 5 s from 60 to 95 °C.

ELISA was used to measure the levels of TNF-α and IL-1β in mouse liver tissue. Total protein was extracted from liver tissue with a total protein extraction kit (Invitrogen, USA). TNF-α (1:2000 dilutions, CST, USA) and IL-1β antibodies (1:2000 dilutions, RD, USA) were then used as the primary antibodies, and HRP-conjugated goat anti-rabbit IgG (1:10,000 dilutions, CST, USA) was used as the secondary antibody. Tetramethylbenzidine (TMB) was added to each well, and 2 M H_2_SO_4_ was used to stop the reaction. The absorbance of each well was measured at 450 nm.

Western blot analysis was used to determine the effect of the NF-κB signalling pathway on liver fibrosis caused by *Cs*sPLA2. Total protein was extracted from liver tissue with a total protein extraction kit (Invitrogen, USA). A BCA protein assay kit (Transgen, China) was used to measure the protein concentration. Mouse β-actin was used as an internal standard for normalization. SDS-PAGE (12% polyacrylamide gel) was used to separate the proteins, and the proteins were immobilized on a polyvinylidene difluoride membrane. The membrane was blocked with 5% skim milk at room temperature for 2 h before being incubated with primary antibodies (p65 antibody and phospho-p65 antibodies, 1:1000 dilution in 1% BSA, CST, USA) overnight at 4 °C. Afterwards, the membrane was incubated with HRP-conjugated goat anti-rabbit IgG (1:10,000 dilution, CST, USA) for 1 h at room temperature. Finally, Pierce™ ECL Plus Western Blotting Substrate (Thermo, USA) was used to visualize the bands.

### Analysis of the inhibitory effect of an NF-κB signalling pathway inhibitor (BAY 11-7082) on liver fibrosis

Fatty-acid-binding protein (FABP) was another ESP of *Clonorchis sinensis*, and here, FABP was used as a negative control. To evaluate the effect of an NF-κB signalling pathway inhibitor (BAY 11-7082) on the liver fibrosis caused by MBP-*Cs*sPLA2, human HSC cells LX-2 were divided into four groups, and incubated with 25 μg/ml MBP, 25 μg/ml MBP-*Cs*sPLA2, 25 μg/ml FABP or 25 μg/ml MBP-*Cs*sPLA2 + 10 μΜ NF-κB inhibitor for 24 h.

The levels of markers of the NF-κB signalling pathway and HSC activation (TNF-α and collagen III, respectively) were measured by quantitative RT-PCR. The sequences of primers for human TNF-α were 5′-CCC AGG CAG TCA GAT CAT CTT CT-3′ (forward primer) and 5′-ATG AGG TAC AGG CCC TCT GAT-3′ (reverse primer). The sequences of primers for human collagen III were 5′-GGT CCT CCT GGA ACT GCC GGA-3′ (forward primer) and 5′-GAG GAC CTT GAG CAC CAG CGT GT-3′ (reverse primer). The sequences of primers for human β-actin were 5′-GTC CAC CGC AAA TGC TTC TA-3′ (forward primer) and 5′-TGC TGT CAC CTT CAC CGT TC-3′ (reverse primer). Human β-actin was used as the internal standard for normalization.

The supernatant of cells was collected to measure the levels of markers of the NF-κB signalling pathway and HSC activation (TNF-α and collagen III, respectively) by ELISA. TNF-α (1:2000 dilutions, CST, USA) and collagen III antibodies (1:2000 dilutions, Abcam, UK) were then applied as the primary antibodies, and HRP-conjugated goat anti-rabbit IgG was applied as the secondary antibody (1:10,000 dilutions, CST, USA).

### Co-immunoprecipitation assay

We transiently over-expressed GFP-tagged *Cs*sPLA2 and Myc-tagged TM7SF3 in 293T cells for 24 h. Transfected 293T cells were lysed using RIPA lysis buffer (Gibco, USA) and incubated with an anti-PLA2 antibody and protein A-agarose beads (Pierce, USA) overnight at 4 °C. After washing five times with RIPA lysis buffer (Gibco, USA), the beads were eluted with 2× SDS sample buffer and boiled for 8 min at 100 °C. The samples were then analysed by western blot using anti-Myc antibodies (Proteintech, USA).

### GST pull-down assay

The recombinant proteins MBP-*Cs*sPLA2 (1.26 mg) and GST-TM7SF3 (0.5 mg) were incubated with 1 mg glutathione-conjugated resin for 2 h at room temperature. GST and MBP-*Cs*sPLA2, or MBP and GST-TM7SF3, were incubated with glutathione-conjugated resin under the same conditions as negative controls. After incubation, mixtures of two proteins were purified by a single-step glutathione-conjugated resin chromatography (GE, USA) under native conditions following the manufacturer’s protocol. The samples were then analysed by western blot using anti-PLA2 antibody.

### Inhibition of HSCs incubated with MBP-*Cs*sPLA2 in the presence of a TM7SF3 antibody by RT-PCR

To assess whether HSCs incubated with MBP-*Cs*sPLA2 are inhibited when TM7SF3 is blocked, LX-2 cells were divided into three groups and incubated with either 25 μg/ml MBP-*Cs*sPLA2 + anti-TM7SF3 antibody (1:200 dilution), 25 μg/ml MBP-*Cs*sPLA2 or PBS. Quantitative RT-PCR was performed to measure the mRNA levels of collagen I and collagen III, markers of HSC activation. The sequences of primers for human collagen I were 5′-CTT CAC CTA CAG CGT CAC TG-3′ (forward primer) and 5′-GGA TGG AGG GAG TTT ACA GG-3′ (reverse primer). The sequences of primers for human collagen III were 5′-GGT CCT CCT GGA ACT GCC GGA-3′ (forward primer) and 5′-GAG GAC CTT GAG CAC CAG CGT GT-3′ (reverse primer). Human β-actin was used as the internal standard for normalization.

### Statistical analysis

GraphPad Prism 6 software was used to analyse the results, and the differences between the control group and the experimental group were analysed by *t* tests at a significance level of *P* < 0.05.

## Results

### The NF-κB signalling pathway was associated with liver fibrosis in liver tissue of mice injected with MBP-*Cs*sPLA2

**Fig. 1:**
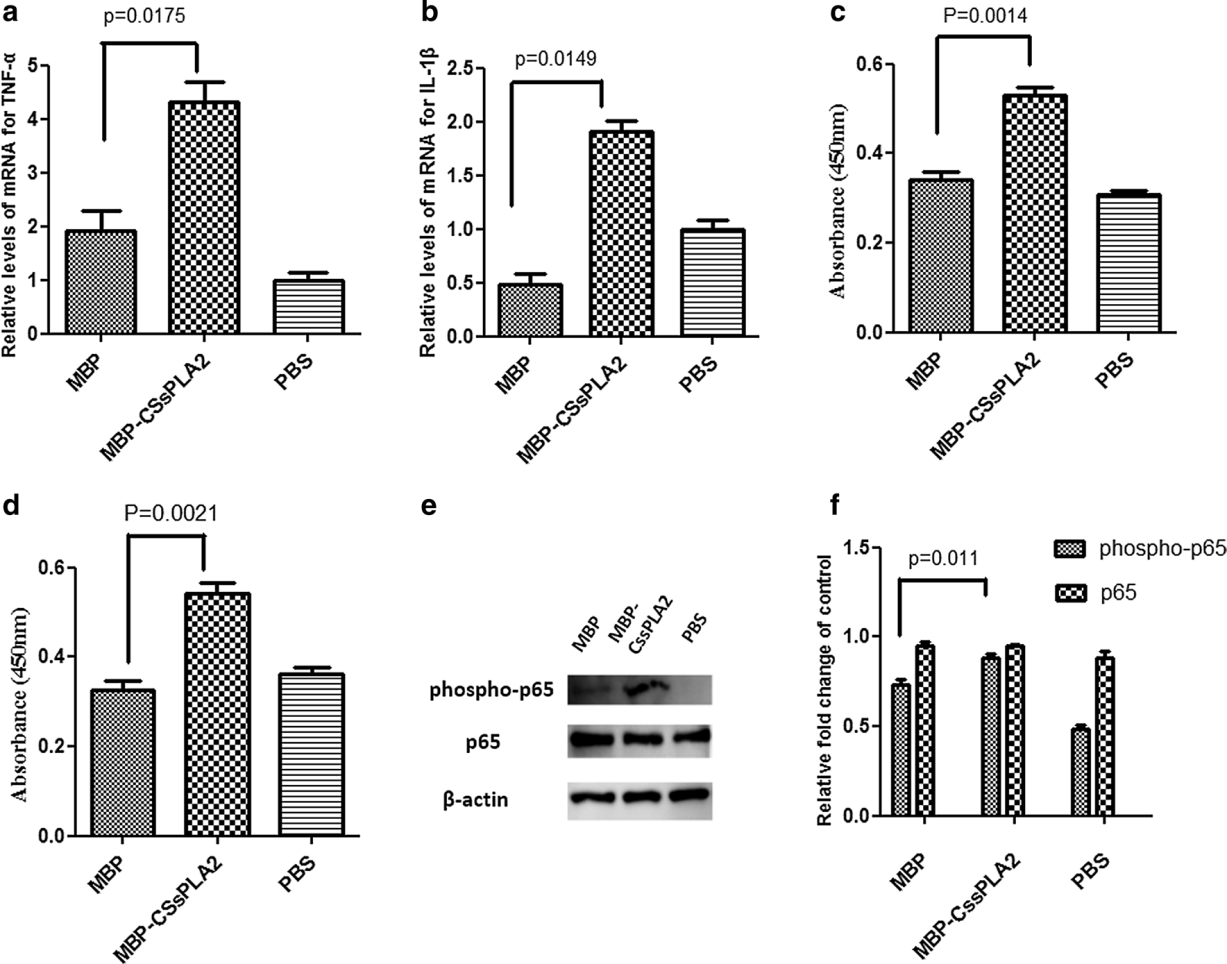
NF-κB signalling pathway is associated with liver fibrosis in the liver tissue of mice injected with MBP-*Cs*sPLA2. **a** The relative mRNA levels for TNF-α of three groups of mice that received abdominal injections of 100 μg PBS, 100 μg MBP or 100 μg MBP-*Cs*sPLA2 (*t*_(4)_ = 3.905, *P* = 0.0175). **b** The relative mRNA levels of IL-1β in the three groups of mice that received abdominal injections of 100 μg PBS, 100 μg MBP or 100 μg MBP-*Cs*sPLA2 (*t*_(4)_ = 4.095, *P* = 0.0149). **c** The absorbance of TNF-α at 450 nm in the three groups of mice that received abdominal injections of 100 μg PBS, 100 μg MBP or 100 μg MBP-*Cs*sPLA2 (*t*_(4)_ = 7.873, *P* = 0.0014). **d** The absorbance of IL-1β at 450 nm in the three groups of mice that received abdominal injections of 100 μg PBS, 100 μg MBP or 100 μg MBP-*Cs*sPLA2 (*t*_(4)_ = 7.052, *P* = 0.0021). **e** Lane 1: liver tissue of a mouse injected with 100 μg MBP; lane 2: liver tissue of a mouse injected with 100 μg MBP-*Cs*sPLA2; lane 3: liver tissue of a mouse injected with PBS. **f** The differences in levels between the control group and the experimental group were determined by quantitation of the western blot results. Unpaired *t* tests were used for statistical analysis (*t*_(4)_ = 4.475, *P* = 0.011)

The level of markers of NF-κB signalling pathway activation in mouse liver tissue were measured by quantitative RT-PCR analysis, and the mRNA of TNF-α and IL-1β were determined. Unpaired *t* tests were performed, which showed that the differences in levels between the group of mice injected with 100 μg MBP and the group injected with 100 μg MBP-*Cs*sPLA2 were significant: *t*_(4)_ = 3.905, *P* = 0.0175 (Fig. 1a) and *t*_(4)_ = 4.095, *P* = 0.0149 (Fig. 1b).

The levels of TNF-α and IL-1β in mouse liver tissue were also measured by ELISA. Both TNF-α and IL-1β were increased in liver tissue of mouse injected with MBP-*Cs*sPLA2. Unpaired *t* tests were performed and showed that the differences in levels between the group injected with 100 μg MBP and the group injected with 100 μg MBP-*Cs*sPLA2 were significant: *t*_(4)_ = 7.873, *P* = 0.0014 (Fig. 1c) and *t*_(4)_ = 7.052, *P* = 0.0021 (Fig. 1d).

The levels of markers of NF-κB signalling pathway activation in mouse liver tissue were measured by western blot. p65 and phospho-p65 were measured in mouse liver tissue. Western blot of the liver tissues of mice injected with 100 μg MBP-*Cs*sPLA2 showed a clear band representing phospho-p65 (Fig. 1e). Representative data from three independent experiments are presented. Significant differences between the group injected with 100 μg MBP and the group injected with 100 μg MBP-*Cs*sPLA2 were revealed by unpaired *t* tests (*t*_(4)_ = 4.475, *P* = 0.011) (Fig. 1f).

### The NF-κB signalling pathway inhibitor (BAY 11–7082) inhibited liver fibrosis in HSCs caused by MBP-*Cs*sPLA2

**Fig. 2:**
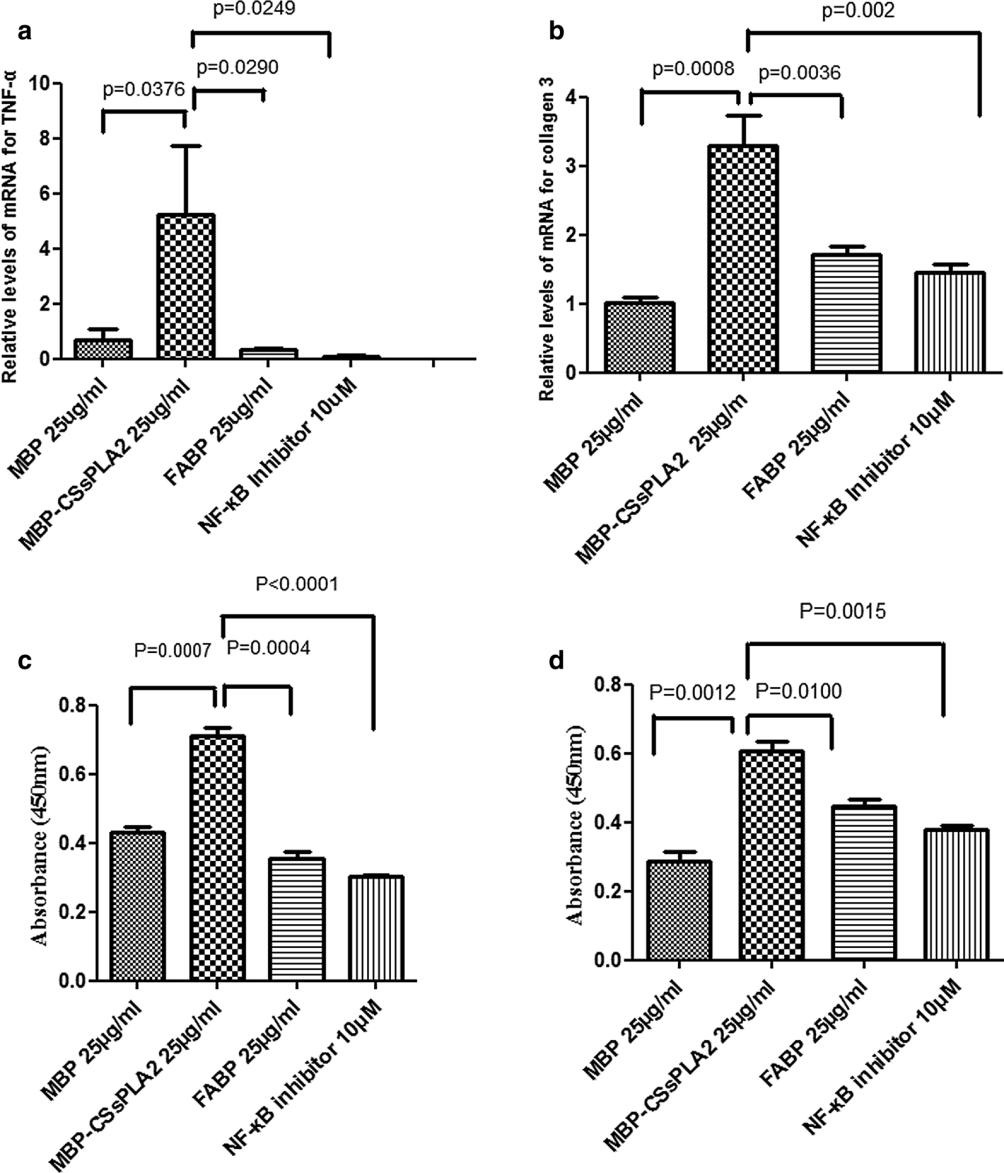
NF-κB signalling pathway inhibitor (BAY 11–7082) inhibited liver fibrosis in HSCs caused by MBP-*Cs*sPLA2. **a** The relative mRNA levels of TNF-α in the four groups of LX-2 cells incubated with 25 μg/ml MBP, 25 μg/ml MBP-*Cs*sPLA2, 25 μg/ml FABP or 25 μg/ml MBP-*Cs*sPLA2 + 10 μΜ NF-κB inhibitor. **b** The relative mRNA levels of collagen III in the four groups of LX-2 cells incubated with 25 μg/ml MBP, 25 μg/ml MBP-*Cs*sPLA2, 25 μg/ml FABP or 25 μg/ml MBP-*Cs*sPLA2 + 10 μΜ NF-κB inhibitor. **c** The absorbance of TNF-α at 450 nm in the four groups of LX-2 cells incubated with 25 μg/ml MBP, 25 μg/ml MBP-*Cs*sPLA2, 25 μg/ml FABP or 25 μg/ml MBP-*Cs*sPLA2 + 10 μΜ NF-κB inhibitor. **d** The absorbance of collagen III at 450 nm in the four groups of LX-2 cells incubated with 25 μg/ml MBP, 25 μg/ml MBP-*Cs*sPLA2, 25 μg/ml FABP or 25 μg/ml MBP-*Cs*sPLA2 + 10 μΜ NF-κB inhibitor

The levels of markers of NF-κB signalling pathway and HSC activation (TNF-α and collagen III, respectively) in HSCs were tested by quantitative RT-PCR analysis. Unpaired *t* tests were performed, and it was found that the differences in levels between the group of cells incubated with 25 μg/ml MBP-*Cs*sPLA2 and the group incubated with 25 μg/ml FABP were significant: *t*_(4)_ = 3.335, *P* = 0.0290 (Fig. 2a) and *t*_(4)_ = 6.113, *P* = 0.0036 (Fig. 2b). Additionally, the differences in levels between the group of cells incubated with 25 μg/ml MBP-*Cs*sPLA2 and the group incubated with 25 μg/ml MBP were significant: *t*_(4)_ = 3.060, *P* = 0.0376 (Fig. 2a) and *t*_(4)_ = 9.043, *P* = 0.0008 (Fig. 2b), and the differences between the group of cells incubated with 25 μg/ml MBP-*Cs*sPLA2 and the group incubated with 25 μg/ml MBP-*Cs*sPLA2 + 10 μΜ NF-κB inhibitor (BAY 11–7082) were significant: *t*_(4)_ = 3.502, *P* = 0.0249 (Fig. 2a) and *t*_(4)_ = 7.157, *P* = 0.002 (Fig. 2b).

The levels of TNF-α and collagen III in HSCs were also measured by ELISA. Unpaired *t* tests were performed, and it was found that the differences in levels between the group of cells incubated with 25 μg/ml MBP-*Cs*sPLA2 and the group incubated with 25 μg/ml FABP were significant [*t*_(4)_ = 11.03, *P* = 0.0004 (Fig. 2c) and *t*_(4)_ = 4.606, *P* = 0.0100 (Fig. 2d)]. Furthermore, the differences in levels between the group of cells incubated with 25 μg/ml MBP-*Cs*sPLA2 and the group incubated with 25 μg/ml MBP were significant [*t*_(4)_ = 9.341, *P* = 0.0007 (Fig. 2c) and *t*_(4)_ = 8.136, *P* = 0.0012 (Fig. 2d)], and the difference between the group of cells incubated with 25 μg/ml MBP-*Cs*sPLA2 and the group incubated with 25 μg/ml MBP-*Cs*sPLA2 + 10 μΜ NF-κB inhibitor (BAY 11-7082) were significant [*t*_(4)_ = 15.81, *P* < 0.0001 (Fig. 2c) and *t*_(4)_ = 7.767, *P* = 0.0015 (Fig. 2d)].

### The interaction between *Cs*sPLA2 and TM7SF3 was confirmed by co-immunoprecipitation

**Fig. 3:**
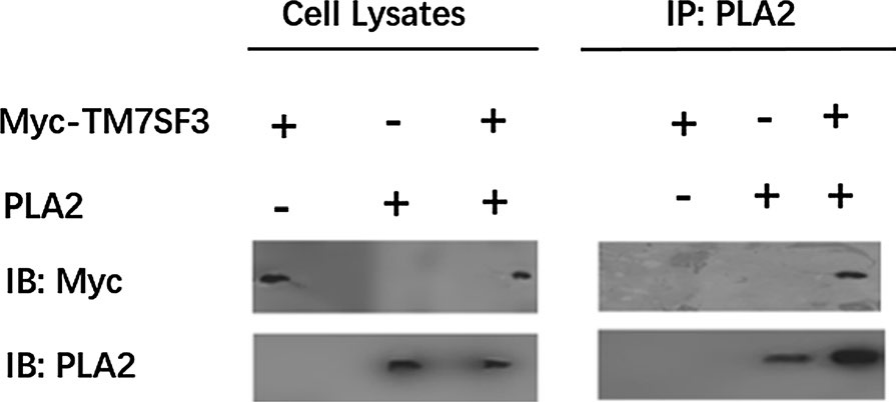
Confirmation of the interaction between CssPLA2 and TM7SF3 by co-immunoprecipitation. The interaction between GFP-tagged *Cs*sPLA2 and Myc-tagged TM7SF3 was assessed by co-IP followed by western blot using anti-PLA2 or anti-Myc antibodies. Tagged proteins were over-expressed in 293T cells by transient transfection. + and – indicate that the recombinant plasmid was or was not transfected into 293T cells, respectively. CssPLA2 pulled down TM7SF3

To analyse the interaction between *Cs*sPLA2 and TM7SF3, GFP-tagged *Cs*sPLA2 and Myc-tagged TM7SF3 were transiently over-expressed in 293T cells. Co-immunoprecipitation revealed that *Cs*sPLA2 pulled down TM7SF3 (Fig. 3).

### The interaction between *Cs*sPLA2 and TM7SF3 was confirmed by GST pull-down

**Fig. 4:**
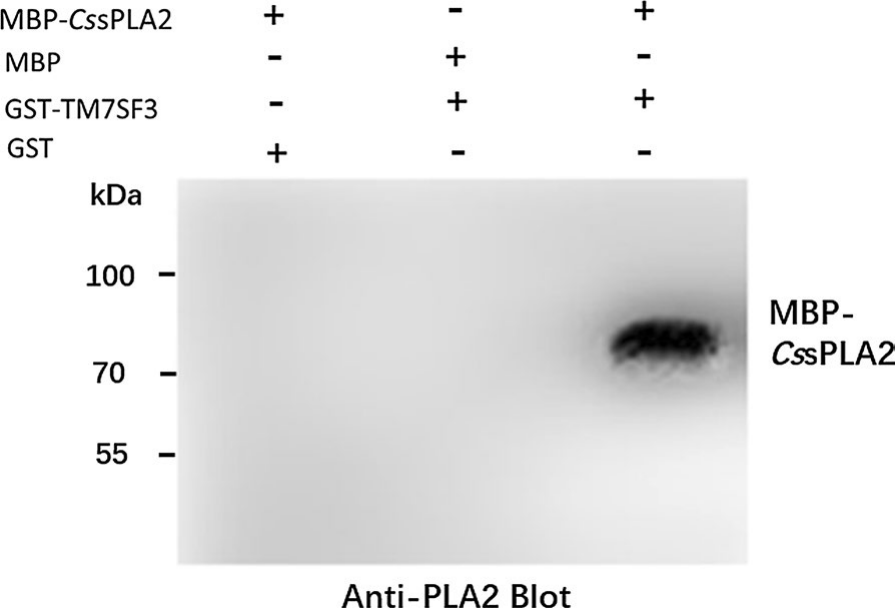
Confirmation of the interaction between *Cs*sPLA2 and TM7SF3 by GST pull-down. The interaction between MBP-tagged *Cs*sPLA2 and GST-tagged TM7SF3 was assessed by GST pull-down followed by western blot using an anti-PLA2 antibody. GST- and MBP-tagged *Cs*sPLA2 or MBP- and GST-tagged TM7SF3 were used as negative controls. TM7SF3 pulled down *Cs*sPLA2

Additionally, we over-expressed MBP-tagged *Cs*sPLA2 and GST-tagged TM7SF3 in *E. coli*. GST pull-down assay revealed that TM7SF3 pulled down *Cs*sPLA2 (Fig. 4).

### TM7SF3 antibody inhibited the activation of HSCs incubated with MBP-*Cs*sPLA2 by blocking TM7SF3

**Fig. 5:**
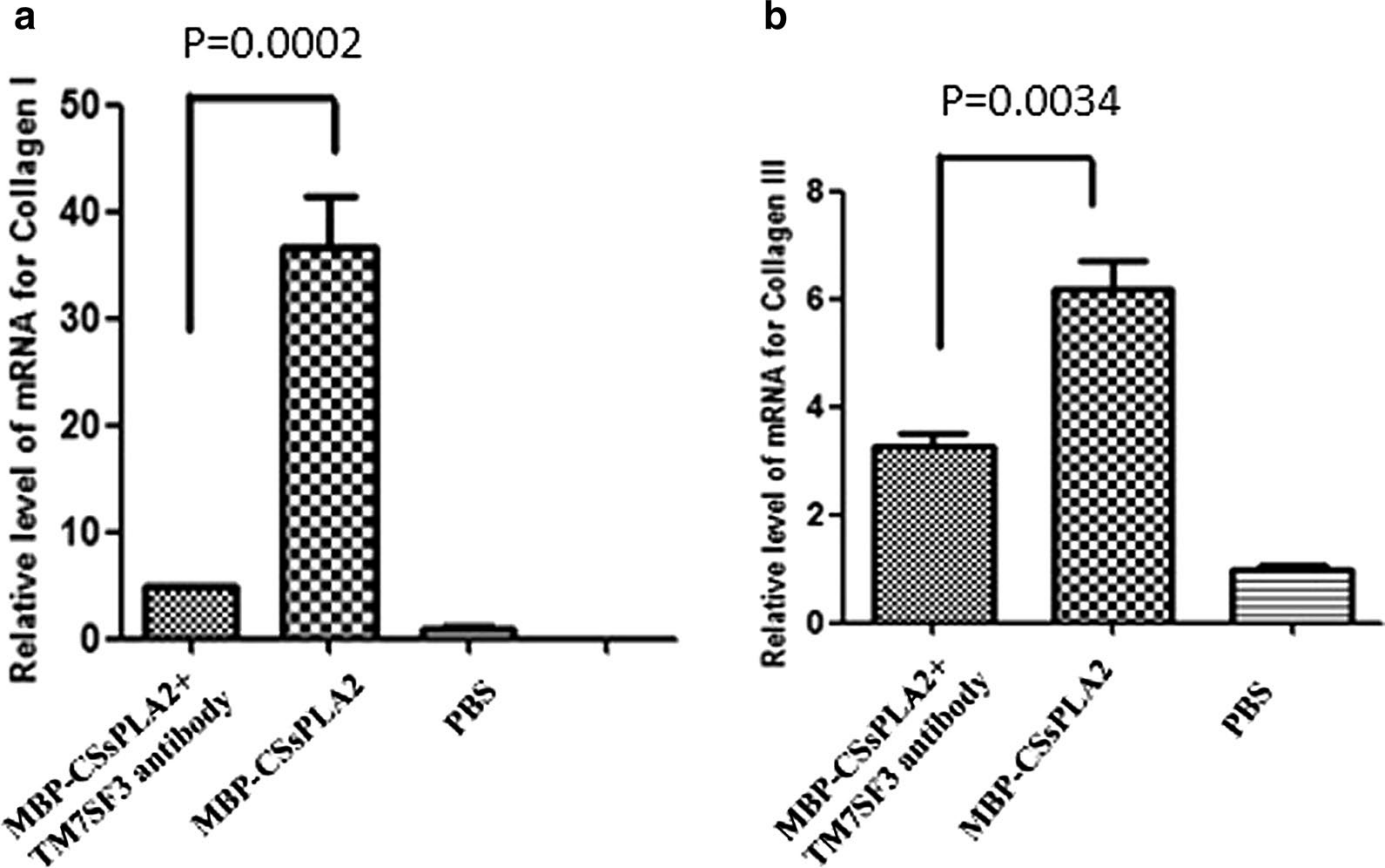
TM7SF3 antibody inhibited the activation of HSCs incubated with MBP-*Cs*sPLA2 by blocking TM7SF3. The mRNA levels of collagen I and collagen III in the group of LX-2 cells incubated with 25 μg/ml MBP-*Cs*sPLA2 + anti-TM7SF3 antibody were lower than those in the group of cells incubated with 25 μg/ml MBP-*Cs*sPLA2. **a** Measurement of collagen I mRNA levels in HSCs treated with an anti-TM7SF3 antibody (*t*_(4)_ = 12.81, *P* = 0.0002). **b** Measurement of collagen III mRNA levels in HSCs treated with an anti-TM7SF3 antibody (*t*_(4)_ = 6.245, *P* = 0.0034)

The levels of the HSC activation markers collagen I and collagen III were measured by quantitative RT-PCR, and the results revealed that since TM7SF3 plays important roles in the activation of HSCs, blocking TM7SF3 inhibited activation of HSCs by MBP-*Cs*sPLA2. Unpaired *t* tests were performed, and it was found that the differences in levels between the group incubated with 25 μg/ml MBP-*Cs*sPLA2 + anti-TM7SF3 antibody and the group incubated with 25 μg/ml MBP-*Cs*sPLA2 were significant [*t*_(4)_ = 12.81, *P* = 0.0002 (Fig. 5a) and *t*_(4)_ = 6.245, *P* = 0.0034 (Fig. 5b)].

## Discussion

NF-κ B signalling regulates activation or apoptosis of HSCs by regulating inflammatory factors such as TNF-α. Expression of NF-κB signalling markers is upregulated in liver fibrosis [18–20]. Therefore, we hypothesized that *Cs*sPLA2 may activate HSCs to cause liver fibrosis through activation of the NF-κB signalling pathway and that the receptor of *Cs*sPLA2, TM7SF3, plays an important role in this process.

This study will contribute to elucidating the mechanism of HSC activation by *Cs*sPLA2 and clarify its role in the process of liver fibrosis caused by *Clonorchis sinensis* infection. Additionally, interfering with the signalling pathway involved in HSC activation by *Cs*sPLA2 may be a potential treatment strategy for liver fibrosis caused by *Clonorchis sinensis* infection.

*Cs*sPLA2-induced activation of human HSCs may involve multiple pathways. For example, the activation of the NF-κB signalling pathway is closely associated with liver fibrosis [21, 22]. The levels of phosphorylated NF-κB were obviously increased in the livers of mice infected with *Clonorchis sinensis* [23]*.* The release of cytokines such as TNF-α and IL-1β [24–27] is closely related to the activation of the NF-κB signalling pathway. The NF-κB signalling pathway is associated with a variety of inflammatory responses, such as TNF-α secretion, which regulates the activation or apoptosis of HSCs during liver fibrosis. The levels of TNF-α, IL-1β, phospho-p65 and other NF-κB signalling markers were increased in the progression of liver fibrosis [18–20]. *Cs*sPLA2 is an important ESP of *Clonorchis sinensis*. A multitude of reports have shown that the ESPs of *Clonorchis sinensis* promote the release of cytokines and induce inflammation [24–27]. *Cs*ESPs are the main causes of liver fibrosis caused by *Clonorchis sinensis*, which can induce HSC activation and proliferation and lead to pathological changes in biliary epithelial cells [28]. Therefore, activation of the NF-κB signalling pathway and cytokine release play important roles in the progression of liver fibrosis induced by *Clonorchis sinensis* infection [29–32]. Based on the above findings, it can be concluded that *Cs*sPLA2-induced activation of HSCs is not the result of the direct action of a single JNK signalling pathway on HSCs, but may also be caused by the NF-κB signalling pathway.

It is generally believed that the activation of signalling pathways is caused by the binding of proteins to their receptors on the cell surface [33–36]. TM7SF3 is located on the surface of HSCs and plays an important role in hepatic fibrosis caused by *Cs*sPLA2. Therefore, TM7SF3, which is the receptor of *Cs*sPLA2, contributes substantially to liver fibrosis caused by *Cs*sPLA2.

## Conclusion

The NF-κB signalling pathway is involved in the activation of HSCs by *Cs*sPLA2. The expression level of the marker of NF-κB signalling pathway activation, phospho-p65, is upregulated in the context of liver fibrosis. Additionally, TM7SF3, the receptor of *Cs*sPLA2, which is located on the surface of HSCs, is related to liver fibrosis caused by *Cs*sPLA2, and inhibition of TM7SF3 with a TM7SF3 antibody relieves liver fibrosis caused by *Cs*sPLA2.

## Data Availability

The nucleic acid sequence of *Cs*sPLA2 supporting the conclusions of this article is available in the GenBank repository (accession numbers: DQ 974199) at https://www.ncbi.nlm.nih.gov/genbank/. The nucleic acid sequence of TM7SF3 supporting the conclusions of this article is available in the Gene repository (Gene ID: 51768) at https://www.ncbi.nlm.nih.gov/gene/51768.

## Abbreviations

*Cs*: *Clonorchis sinensis*
*Css*PLA2: Secreted phospholipase A2 of *Clonorchis sinensis*
*s*PLA2: Secreted phospholipase A2
ESPs: Excretory and secretory products
MBP: Maltose-binding protein
TM7SF3: Transmembrane 7 superfamily member 3
